# A Smartphone-Smartcard Platform for Implementing Contingency Management in Buprenorphine Maintenance Patients With Concurrent Stimulant Use Disorder

**DOI:** 10.3389/fpsyt.2021.778992

**Published:** 2021-12-07

**Authors:** Anthony DeFulio, Joshua Furgeson, Hayley D. Brown, Shawn Ryan

**Affiliations:** ^1^Department of Psychology, Western Michigan University, Kalamazoo, MI, United States; ^2^Statistical Consultant, Boston, MA, United States; ^3^BrightView Health, Inc., Cincinnati, OH, United States

**Keywords:** digital health (eHealth), opioid use disorder, cocaine, methamphetamine, incentive-based intervention, medication-assisted treatment (MAT), stimulant use disorder

## Abstract

**Background and Objectives:** Opioid agonist pharmacotherapies are effective in the treatment of opioid use disorder (OUD) but concurrent stimulant use is common and can lead to relapse and treatment drop out. Contingency management in combination with opioid agonist pharmacotherapy has broad beneficial effects in polysubstance users, including promoting drug abstinence and treatment retention, but clinic-based implementation can be burdensome. The present study was conducted to evaluate a contingency management intervention delivered *via* a smartphone-smartcard platform in OUD patients who had concurrent stimulant use disorder.

**Methods:** Retrospective comparison of (*n* = 124) patients; half received the contingency management intervention and half were matched controls. Drug use and clinic attendance outcomes over four consecutive 30-day periods were analyzed with regression.

**Results:** The intervention group showed consistently higher rates of drug abstinence and clinic attendance which were significant at the latter two timepoints.

**Discussion:** Smartphone-smartcard platforms can facilitate dissemination of contingency management by surmounting or obviating key barriers to adoption. They appear to be convenient for all stakeholders, are easy to use, and facilitate high-fidelity implementation. Delivering contingency management *via* a smartphone-smartcard platform produces effects consistent with those observed when the intervention is delivered with substantially costlier and more burdensome in-person procedures.

## Introduction

Opioid agonist pharmacotherapies such as buprenorphine and methadone are effective in the treatment of opioid use disorder. However, for people enrolled in buprenorphine maintenance pharmacotherapy, concurrent stimulant use is associated with higher rates treatment dropout (1, 2). There is currently no effective medication for the treatment of stimulus use disorder. In a recent systematic review of reviews, contingency management was the only supported treatment for stimulant use disorder (3). Contingency management typically entails the provision of material incentives (e.g., vouchers exchangeable for goods or services) contingent upon submission of drug toxicology tests that indicate recent drug abstinence. Adding contingency management to pharmacotherapy for opioid use disorder has significantly and robustly improved outcomes in polydrug users (4).

Despite its success in clinical trials, adoption of contingency management has been slow among outpatient treatment providers. Barriers have been studied extensively, and include a lack of training and expertise, a lack of time for implementing the procedures, a lack of infrastructure required to conduct the program, and the lack of a stable means of funding the program costs (5). Save for costs, these barriers are wholly obviated by delivering the intervention *via* a smartphone-smartcard platform that automates all aspects of intervention management. This delivery system for contingency management intervention has been shown to be effective in promoting smoking cessation (6), alcohol abstinence (7), and in promoting drug abstinence and clinic attendance in people receiving outpatient treatment for opioid use disorder at an inner-city clinic (8).

Given the high risk of treatment dropout for buprenorphine patients with concurrent stimulant use disorder, the historic success of contingency management for patients with similar profiles, and the need for a scalable, rapidly disseminable platform to enhance the clinical impact of contingency management, we sought to evaluate the efficacy of a smartphone-smartcard contingency management platform for increasing treatment attendance and drug abstinence in buprenorphine patients with concurrent stimulant use disorder.

## Materials and Methods

### Sample

Intervention participants were recruited from a BrightView Health Center located in Cincinnati, Ohio. Enrollees were required to own their own Android or iOS smartphone. Overall, 108 patients enrolled in the smartphone-smartcard contingency management intervention. The present analysis was restricted to enrollees with an opioid use disorder who had concurrent stimulant (i.e., cocaine and/or methamphetamine) use disorder (*n* = 67). These participants were retrospectively matched, blind as to outcomes, to control patients at another BrightView clinic in the same city that did not offer the smartphone-smartcard contingency management intervention. All participants in both groups were receiving similar treatment for their substance use disorders at BrightView clinics. Matched controls (1) completed a urinary drug toxicology test at the clinic on or before the day the intervention patient started the contingency management intervention, (2) were enrolled at the clinic on the day the intervention patient started the contingency management intervention, (3) had the same primary diagnosis (e.g., Opioid Use Disorder), and (4) had the same American Society of Addiction Medicine (ASAM) Level of Care at the time of clinic enrollment (e.g., outpatient vs. intensive outpatient).

When multiple control patients met all the criteria, the control patient who first entered treatment closest to the participant's start was chosen to improve the match on treatment timeframe and duration. Five participants were excluded due to a lack of appropriately matched control patients resulting in a final sample of 124 patients (62 matched patient-pairs), all of whom were included in the clinical characterization and main outcome analyses.

### Intervention

This study involved the pilot implementation of a smartphone-smartcard platform developed by DynamiCare Health, Inc. (Boston, MA) described in detail elsewhere (8). The contingency management intervention provided appointment reminders with smartphone GPS monitoring, cognitive behavioral therapy readings with exercises and comprehension questions, and up to $100 per month in monetary incentives for these and for abstinent substance tests. Rewards were paid promptly and automatically *via* a smart debit card that offered numerous protections spending that was inconsistent with the goals of treatment.

### Analysis

Main outcome analyses were conducted for attendance and urine samples consistent with illicit drug abstinence and medication adherence requirements, which were individualized based on the needs of each patient. Group outcomes were compared in four consecutive 30-day blocks. Attendance was calculated as the percentage of all scheduled appointments attended for each 30-day block and was analyzed with ordinary least squares regression. Logistic regression was used to analyze the percentage of urine samples consistent with illicit drug abstinence and medication adherence. Any missing outcome data were imputed as the undesirable outcome for the analysis. To control for possible confounders and an important predictor, the regression models included covariates for new patient status, cocaine use disorder diagnosis, and baseline urine sample result.

For continuous demographic and clinical characteristics, *p*-values were estimated using a two-sample *t*-test. For dichotomous characteristics, *p*-values were estimated using Fisher's exact *p*. Intervention group participants were classified as new patients if they enrolled in in the contingency management intervention within 5 days of starting treatment. Comparison patients enrolled in treatment at the clinic within 5 days of their matched intervention participant starting the smartphone-smartcard contingency management intervention.

## Results

In terms of demographics, there were no significant differences between groups. Combining the groups shows that the sample was 52% female and 89% white, with an average age of 38 (*SD* = 9.2). Further, 79% of the participants had completed high school, 48% were unemployed, and 11% were married (for these characteristics, some clinic records were incomplete, with the number of missing values ranging from 3 to 19 depending on the measure).

Clinical characterization of the sample revealed two significant baseline differences: the intervention group contained 37% new patients compared to 15% in the control group (*p* < 0.05), and the intervention group contained 74% patients with a cocaine use disorder diagnosis compared to 48% of the control patients (*p* < 0.01). In both groups, 95% of participants had opioid use disorder as their primary diagnosis. Similarly, in both groups 60% of participants were enrolled in a level 1 outpatient program, 37% were enrolled in an intensive outpatient program, and 3% were enrolled in continuing care. In the intervention group, 24% of participants' baseline urine sample was consistent with clinic requirements, compared to 21% of participants in the comparison group.

**Figure 1:**
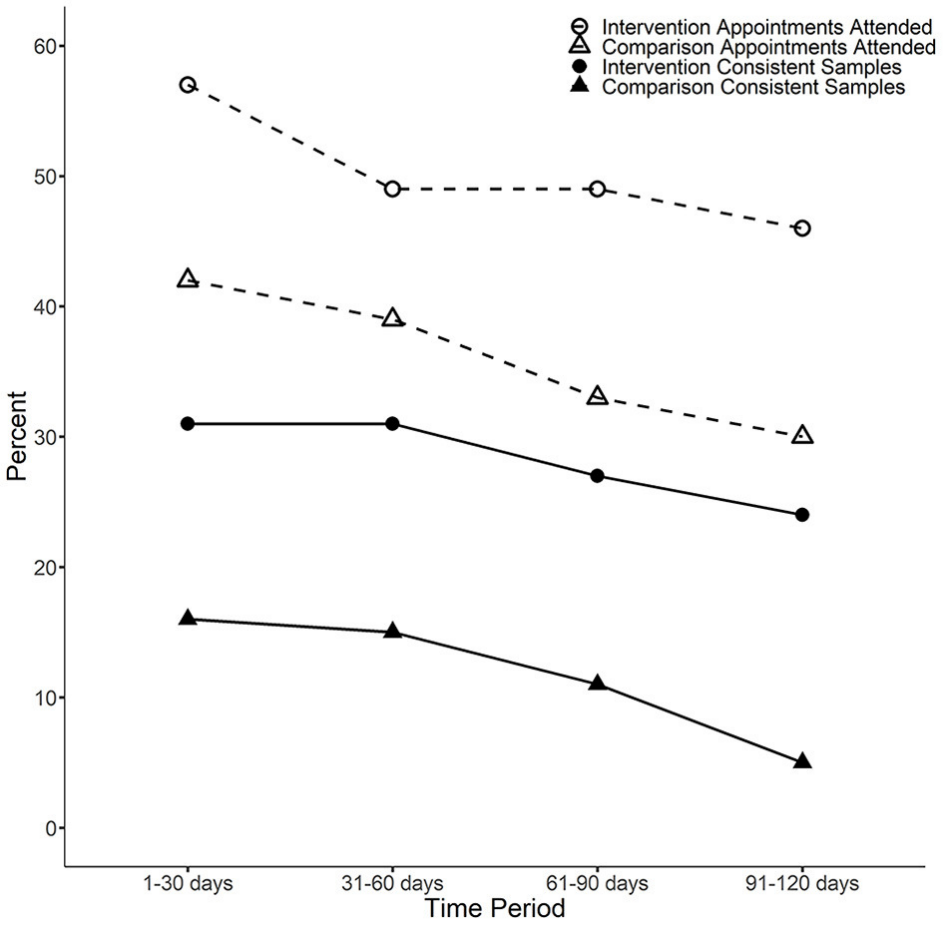
The percentage of consistent samples and appointment attendance rate by condition and time block. Consistent urine samples: For the third and fourth time periods, a Fisher's exact *p* test indicates the average difference between intervention and comparison patients is statistically significant (*p* < 0.05). For the first time period (1–30 days), the *p*-value is *p* = 0.089 and for the second time period (31–60 days) the *p*-value is *p* = 0.052. Attendance: For the first, third and fourth time periods, a two-sample *t*-test indicates the average difference between intervention and comparison patients is statistically significant (*p* < 0.05). For the second time period (31–60 days), the *p*-value is *p* = 0.062.

The results for the consistent urine sample and attendance record analyses are shown in Figure 1 and Table 1. Participants in the intervention group were significantly more likely to attend appointments at all time points, and more likely to submit consistent urine samples at the third and fourth time points. Both groups showed declines in consistent urine samples and attendance over time, but the declines were more substantial in the control. A sensitivity analysis only including matched-pairs with complete data (i.e., treating missing urine or attendance samples as missing instead of imputing as inconsistent or zero) typically estimated similar effects, although the smaller sample sizes resulted in less statistical power.

**Table 1:** Consistent urine tests and appointment attendance.

	**1–30 days**	**31–60 days**	**61–90 days**	**91–120 days**
Consistent urine test outcomes				
Intercept	−2.35** (0.51)	−2.40** (0.50)	−2.55** (0.54)	−3.32** (0.70)
Intervention	0.96* (0.55) [2.60]	0.98* (0.53) [2.68]	1.46** (0.60) [4.31]	2.24** (0.76) [9.43]
New treatment patient	0.34 (0.54)	0.08 (0.54)	−1.01 (0.66)	−1.10 (0.72)
Cocaine diagnosis	−0.27 (0.52)	0.06 (0.52)	−0.23 (0.56)	−0.32 (0.63)
Consistent baseline test	2.21** (0.51)	1.82** (0.50)	1.94** (0.53)	1.58** (0.59)
Appointment attendance rate outcomes				
Intercept	48.69** (4.24)	44.32** (5.01)	35.85** (5.06)	35.18** (5.54)
Intervention	18.93** (4.81)	14.065* (5.68)	20.83** (5.73)	20.07** (6.27)
New treatment patient	−8.71 (5.32)	−10.82* (6.28)	−17.54** (6.34)	−14.86** (6.95)
Cocaine diagnosis	−8.81* (4.76)	−6.28 (5.62)	−2.10 (5.70)	−3.41 (6.21)
Consistent baseline test	−3.38 (5.40)	−2.33 (6.38)	2.60 (6.44)	−5.14 (7.05)
Adjusted *R*^2^	0.09	0.03	0.10	0.06

* *p < 0.10*,

** *p < 0.05. Consistent urine tests mean that the patient was negative for all tested substances and positive for expected prescribed medications. The logistic regression coefficient is the first number listed in each cell, with standard errors in parentheses. The odds ratio is in brackets and is the odds of a consistent test for intervention group patients over the odds of a consistent test for comparison patients (i.e., numbers >1 indicate a positive intervention impact). For example, between 61 and 90 days, the odds of an intervention patient having a consistent urine test are 4.31 the odds of a comparison patient having a consistent urine test (p < 0.05). The coefficients indicate the increase in percentage attendance (e.g., between 61 and 90 days, intervention patients had a 20.83% point higher rate of appointment attendance)*.

## Discussion

The present study shows that contingency management delivered *via* a smartphone-smartcard platform can improve drug use and clinic attendance outcomes among patients with concurrent opioid and stimulant use disorders when used as an adjunct to care in an outpatient buprenorphine maintenance program. The present finding is broadly consistent with prior contingency management studies in general (3–5), and with prior studies of the same smartphone-smartcard platform for delivery contingency management intervention (6–8). This is a timely finding, as the Office of National Drug Control Policy (ONDCP) top priority for 2021 is, “Expanding access to evidence-based treatment”, and specific actions described by the ONDCP toward this end include, “Identify and address policy barriers related to contingency management interventions (motivational incentives) for stimulant use disorder”, and “Explore reimbursement for motivational incentives and digital treatment for addiction, especially stimulant use disorder” (9).

The most important limitation of the present study is the possible selection bias, as patients chose whether to enroll in the treatment. Another key limitation is that the retrospective design used in this study is not as strong as a randomized controlled trial. Nevertheless, widespread dissemination of contingency management for the treatment of polysubstance use is urgent and digital platforms offer dissemination potential that cannot be matched by training programs designed to enable outpatient providers to offer clinic-based contingency management services directly. One of the key advantages of digital platforms is that they allow for the delivery of high-fidelity contingency management. Another advantage is that commercial and Medicaid payers are beginning to support this form contingency management, which provides a pathway to addressing the issue of cost as a barrier to adoption of contingency management.

Future studies should explore combinations of drug abstinence and medication adherence contingencies, and seek to explicitly evaluate long-term treatment retention and outcomes. A recent review highlighted the success of contingency management in producing good outcomes 1-year post-treatment (10), but whether similar outcomes can be achieved with a remote digital delivery platform remains unknown. Another potential advantage of delivering contingency management *via* a remote digital platform is that a wide variety of patient behaviors can be measured and used to predict lapses and treatment dropout, and provide immediate therapeutic response (e.g., *via* peer-recovery coaching) just-in-time, in an attempt to support patients at times of elevated risk.

## Data Availability Statement

The raw data supporting the conclusions of this article will be made available by the authors, without undue reservation.

## Ethics Statement

The studies involving human participants were reviewed and approved by Sterling Institutional Review Board. Written informed consent for participation was not required for this study in accordance with the national legislation and the institutional requirements.

## Author Contributions

SR was principally responsible for overseeing data collection. JF was principally responsible for conducting the statistical analysis. AD was principally responsible for all aspects of manuscript preparation. All authors designed the study together and read and approved the final version of the manuscript.

## Funding

The study was supported by a grant from the Ohio Opioid Technology Challenge awarded to DynamiCare Health Inc., and BrightView Health, Inc. The funders had no role in the design of the study, the analysis of the data, or the preparation of the manuscript.

## Conflict of Interest

AD and JF have served as paid research consultants for DynamiCare Health, Inc. SR is employed by BrightView Health, Inc. The remaining author declares that the research was conducted in the absence of any commercial or financial relationships that could be construed as a potential conflict of interest.

## Publisher's Note

All claims expressed in this article are solely those of the authors and do not necessarily represent those of their affiliated organizations, or those of the publisher, the editors and the reviewers. Any product that may be evaluated in this article, or claim that may be made by its manufacturer, is not guaranteed or endorsed by the publisher.
